# *Atp1b2^Atp1b1^* Knock-In Mice Exhibit a Cone–Rod Dystrophy-Like Phenotype

**DOI:** 10.3390/cells14120878

**Published:** 2025-06-11

**Authors:** Susanne Bartsch, Yevgeniya Atiskova, Stefanie Schlichting, Elke Becker, Maike Herrmann, Udo Bartsch

**Affiliations:** Department of Ophthalmology, Experimental Ophthalmology, University Medical Center Hamburg-Eppendorf, 20246 Hamburg, Germany; s.bartsch@uke.de (S.B.); y.atiskova@uke.de (Y.A.); st.schlichting@uke.de (S.S.); e.becker@uke.de (E.B.); ma.herrmann@uke.de (M.H.)

**Keywords:** ATP1A3, ATP1B2, bipolar cells, cones, cone–rod dystrophy, degeneration, Na,K-ATPase, photoreceptor cells, retina, retinoschisin, rods, voltage-gated potassium channel

## Abstract

The Na,K-ATPase is a heterodimeric ion pump consisting of various combinations of a catalytic α-subunit (α1, α2, α3, or α4, encoded by *ATP1A1–ATP1A4*) and a β-subunit (β1, β2, or β3, encoded by *ATP1B1–ATP1B3*). We have previously shown that *Atp1b2* knock-out (ko) mice exhibit rapid photoreceptor cell degeneration, whereas *Atp1b2^Atp1b1^* knock-in (ki) mice, which express the β1-subunit instead of the β2-subunit under regulatory elements of the *Atp1b2* gene, exhibit slowly progressive retinal dystrophy. Here, we performed a detailed analysis of the retinal phenotype of the *Atp1b2^Atp1b1^* ki mouse. We found that the number of cone photoreceptor cells in the mutant retinas was significantly reduced by postnatal day 28. The retinas of 4-month-old mice were almost devoid of cones. The early onset and rapid loss of cones was followed by a slowly progressive degeneration of rods. Other retinal cell types were unaffected. Nonradioactive *in situ* hybridization and immunohistochemistry revealed that wild-type photoreceptors expressed β3 and high levels of β2, while *Atp1b2^Atp1b1^* ki photoreceptor cells expressed β3 and low levels of transgenic β1. Additionally, levels of retinoschisin, a secreted retina-specific protein that interacts directly with the β2-subunit, were greatly reduced in mutant retinas. The results demonstrate that the β1-subunit can functionally compensate, at least in part, for the absence of the β2-subunit. The results also show that cones are more susceptible to Na,K-ATPase dysfunction than rods. Taken together, the present study identifies the *Atp1b2^Atp1b1^* ki mutant as a novel animal model of an early-onset and rapidly progressive cone–rod dystrophy.

## 1. Introduction

The Na,K-ATPase is a transmembrane ion pump consisting of a catalytic α-subunit (α1, α2, α3, or α4) and a regulatory β-subunit (β1, β2, or β3) which act as molecular chaperones required for the intracellular transport, functional maturation, and stable integration of the α-subunits into the cell membrane. The αβ heterodimers can associate with members of the FXYD protein family, which stabilize the enzyme and modulate its kinetic properties. The ubiquitously expressed ion pump is essential for cell function and survival. The α-subunit hydrolyzes ATP to actively transport Na^+^ ions out of the cell and K^+^ ions into the cell, thereby creating an electrochemical gradient that is critical for regulating cell volume, muscle contractility, and neuronal excitability, and for providing energy to secondary transport systems that translocate ions, nutrients, and neurotransmitters across the cell membrane. The enzyme also functions as an important signal transducer [1,2,3,4,5,6,7]. While any α-subunit can combine with any β-subunit to form a functional Na,K-ATPase [1,8], the expression of different heterodimers *in vivo* is developmentally regulated in a cell type- and tissue-specific manner [1,9,10].

The β2-subunit was originally characterized as a Ca^2+^-independent cell adhesion molecule predominantly expressed in glial cells. Therefore, the protein was named adhesion molecule on glia (AMOG) [11]. Cell culture experiments have shown that AMOG mediates neuron-astrocyte adhesion, promotes granule cell migration along Bergmann glial processes in cerebellar explant cultures, and supports neurite outgrowth of cerebellar and hippocampal neurons [11,12]. While AMOG has been reported to mediate adhesion via heterophilic trans-interactions with a yet unknown receptor [12,13], recent evidence suggests that it also acts as a homophilic trans-interacting adhesion molecule [14]. Subsequent work has shown that AMOG shares approximately 40% amino acid identity with β1, and is identical to a putative Na,K-ATPase β-subunit, identified in rat brains and human lungs, and termed β2 [15,16]. Indeed, AMOG was found to be tightly associated with the α2-subunit of the Na,K-ATPase, anti-AMOG antibodies increased pump activity in cultured astrocytes [16], and AMOG expressed in *Xenopus* oocytes assembled with either endogenous *Xenopus* α1 or co-expressed *Torpedo* α1 to form functional sodium pumps [17]. Notably, the Na,K-ATPase inhibitor ouabain did not interfere with AMOG-mediated neuron-astrocyte adhesion. Based on the combined results, it was suggested that AMOG (hereafter referred to as β2) links cell-cell interactions with the regulation of ionic homeostasis [16,18].

The function of β2 *in vivo* has been studied in two transgenic mouse lines. *Atp1b2* knock-out (ko; hereafter referred to as *β2* ko) mice developed a severe neurological phenotype and died prematurely during the third postnatal week [18]. These mutant mice developed motor deficits and exhibited significantly enlarged ventricles, swollen astrocytic endfeet, and electron-lucent vacuoles adjacent to blood vessels in several brain regions. Of interest in the context of the present study, *β2* ko mice also exhibited a rapidly progressive apoptotic degeneration of photoreceptor cells [18,19]. The neuropathological abnormalities in the brain and retina of the *β2* ko mouse were attributed to impaired pump activity [18]. In *Atp1b2^Atp1b1^* ki mice (hereafter referred to as *β2*/*β1* ki mice), β2 expression is abolished and replaced by a fusion protein consisting of 18 N-terminal amino acids of the β2-subunit and amino acids 14 to 304 of the β1-subunit [20]. The knocked-in transgene prevented the brain pathology observed in *β2* ko mice. Furthermore, *β2*/*β1* ki mice had a normal life expectancy, indicating that β1 can functionally substitute for the absence of β2. The only pathology found in *β2*/*β1* ki mice was a progressive loss of photoreceptor cells which, however, progressed at a significantly slower rate than in *β2* ko mice. The results indicated that restoration of Na,K-ATPase activity by transgenic β1 was not sufficient to rescue this metabolically highly active cell type from death [20]. Developmental abnormalities consistent with a critical role of β2 as an adhesion molecule *in vivo*, such as ectopically located granule cells in the cerebellar cortex, were not observed in either mouse line [18,20].

Photoreceptor cells in adult mice predominantly express an α3β2 heterodimer [10,21,22]. Interestingly, a recent study has provided evidence that mutations in the *ATP1A3* gene cause autosomal dominant cone–rod dystrophy in humans [23]. In addition, the Na,K-ATPase α3β2 isozyme has been shown to interact directly with retinoschisin, a secreted protein mainly localized on the surfaces of photoreceptor inner segments and bipolar cells. Mutations in *RS1*, the gene encoding retinoschisin, cause X-linked retinoschisis (XLRS), a retinal disease characterized by the splitting of retinal layers and loss of visual acuity [24,25,26,27,28]. Furthermore, the α3β2 isozyme has recently been shown to interact with the voltage-gated potassium (Kv) channel subunits Kv2.1 and Kv8.2 [29]. Notably, mutations in *KCNV2* encoding Kv8.2 cause the retinal disorder cone dystrophy with supernormal rod response (CDSRR) [30,31].

In the present study, we examined the expression of the different β-subunits in wild-type and *β2*/*β1* ki photoreceptor cells, investigated whether cone and rod photoreceptors are differentially affected in *β2*/*β1* ki mice, studied the fate of retinal nerve cell types other than photoreceptors in adult mutants, and analyzed the expression and localization of the α3-subunit, retinoschisin, Kv2.1, and Kv8.2. We observed an early-onset and rapidly progressive loss of cone photoreceptors followed by a slowly progressive degeneration of rod photoreceptors, demonstrating that cones and rods differ in their susceptibility to Na,K-ATPase dysfunction.

## 2. Materials and Methods

### 2.1. Animals

*β2*/*β1* ki mice [20] and wild-type mice were maintained on a C57BL/6J genetic background and housed in a 12-h light/dark cycle with ad libitum access to food and water at the animal facility of the University Medical Center Hamburg-Eppendorf (Hamburg, Germany). The *β2*/*β1* ki mice were bred homozygously. Because we observed no difference in the severity of the retinal phenotype between sexes, males and females were included in the study. Animal experiments were performed in accordance with EU Directive 2010/63/EU and were approved by the Ethics Committee of the Freie und Hansestadt Hamburg (ORG 842 and ORG 1089).

### 2.2. Immunohistochemistry

Retinas were processed for immunohistochemical analysis as previously described [32]. In brief, *β2*/*β1* ki and wild-type mice were euthanized, eyes were immersion-fixed in a phosphate-buffered saline (PBS) containing 4% paraformaldehyde, cryoprotected, frozen, and sectioned at a thickness of 25 µm using a cryostat (CM1950, Leica, Wetzlar, Germany). The sections were blocked in PBS containing 0.1% bovine serum albumin (BSA; Sigma-Aldrich, Deisenhofen, Germany) and 0.3% Triton X-100, incubated with the primary antibodies (Table 1), washed, and incubated with Cy2- or Cy3-conjugated secondary antibodies (1:200; Jackson Immunoresearch Laboratories, West Grove, PA, USA). Cone photoreceptor cells were also labeled using biotinylated peanut agglutinin (PNA; 1:5000; Vector Laboratories, Burlingame, CA, USA) and Cy3-conjugated streptavidin (1:500; Jackson Immunoresearch Laboratories). Cell nuclei were stained with 4′,6-diamidino-2-phenylindole (DAPI; Sigma-Aldrich). All immunostainings were performed on free-floating retinal sections in microwell plates. To compare the expression levels of the α3-subunit, retinoschisin (RS1), and the voltage-gated potassium (Kv) channel subunits Kv2.1 and Kv8.2 in wild-type and mutant retinas, sections from both genotypes were processed in the same well and photographed using the same microscope settings. Retinal ganglion cells (RGCs) were visualized in retinal flatmounts using antibodies against brain-specific homeobox/POU domain protein-3A (BRN-3A) as described [33]. For qualitative documentation of immunohistochemical experiments, z-stacks of identical thickness were acquired for each antigen using an AxioObserverZ.1 microscope equipped with an ApoTome.2 (Zeiss, Oberkochen, Germany).

### 2.3. Retina Thickness and Number of Retinal Neurons

Using an AxioObserver Z.1 microscope, images with an optical section thickness of 0.6 µm were taken from the nasal to the temporal periphery of central retinal sections (i.e., in the plane of the optic nerve head). Using ZEN 2.3 PRO software (Zeiss), the thickness of the total retina, the photoreceptor layer (i.e., the inner and outer segments, the photoreceptor cell bodies, and the outer plexiform layer), and the inner nuclear layer were measured at nine equidistant positions in both the nasal and temporal retinas. Rows of photoreceptor cell nuclei were counted at these same positions. We counted cone arrestin- or m+s-opsin-labeled cone photoreceptor cells, protein kinase C alpha (PKCα)-positive rod bipolar cells, and secretagogin (SCGN)-positive cone bipolar cells with clearly visible DAPI-labeled nuclei over the entire length of the sections [35]. The density of BRN-3A-positive RGCs was determined in retinal flatmounts as described [33]. Thickness measurements and photoreceptor counts were performed on 6 *β2*/*β1* ki and 6 wild-type mice of each age (i.e., postnatal day (P) 14, P28, P56, P112, and P168). The densities of RGCs, rod bipolar cells, and cone bipolar cells were determined in 168-day-old mutant mice and age-matched wild-type mice (n = 6 of each genotype). The statistical analysis of thickness measurements and cell densities was performed with GraphPad Prism V5.02 software (GraphPad Software, San Diego, CA, USA) using the two-way ANOVA followed by a Bonferroni post hoc test. The statistical analysis of rod bipolar cell, cone bipolar cell, and RGC densities was performed using the Student’s *t*-test.

### 2.4. In Situ Hybridization

A 1063 base pair (bp) fragment of *Atp1b1* consisting of 502 bp 3′-translated and 561 bp untranslated sequence, a 760 bp fragment of *Atp1b2* encoding exons II to VII, and a 1052 bp fragment of *Atp1b3* consisting of 553 bp 3′-translated and 499 bp untranslated sequence were PCR amplified from adult mouse brain cDNA and cloned into the pCR^TM^II-Topo^®^ vector (Invitrogen, Thermo Fisher Scientific, Waltham, MA, USA). Digoxigenin-labeled antisense and sense cRNA probes were generated by *in vitro* transcription of linearized plasmids using the T7 and SP6 polymerases, respectively. *In situ* hybridization experiments were performed on 14 µm thick cryostat sections of 2-month-old wild-type and age-matched *β2*/*β1* ki retinas, as previously described [36]. To control for the specificity of the signals obtained with the antisense cRNA probes, retinas were hybridized with the *Atp1b1* or *Atp1b3* sense probes. Hybridization of *β2*/*β1* ki retinas with the *Atp1b2* antisense cRNA probe served as a further negative control.

### 2.5. Western Blot

Retinas of two-month-old β2/β1 ki and age-matched wild-type mice were homogenized in a lysis buffer containing 50 mM Tris-HCl (pH 7.5), 150 mM NaCl, 1% Triton X-100, and a protease inhibitor cocktail (cOmplete™ Mini; Roche, Basel, Switzerland); incubated on ice for 30 min; and centrifuged at 20,000× *g* for 10 min. The protein concentrations of the resulting supernatants were determined using a bicinchoninic acid assay (Pierce™ BCA Protein Assay; Thermo Fisher Scientific), with bovine serum albumin (BSA) serving as the standard. Total protein extracts were separated by SDS-PAGE and blotted onto PVDF membranes. Membranes were blocked with 3.5% nonfat dry milk, incubated overnight at 4 °C with an anti-retinoschisin antibody, washed three times, and incubated with an IRDye^®^ 800CW donkey anti-rabbit secondary antibody (1:20,000; LI-COR Biosciences, Lincoln, NE, USA) in Tris-buffered saline containing 0.2% Tween 20 for 1 h at room temperature. To control for loading, the membranes were stained with Revert^TM^ 700 Total Protein Stain (TPS; LI-COR Biosciences). Immunolabeled bands were visualized using the LI-COR Odyssey^®^ Fc Imaging System. Signal intensities were quantified using Empiria Studio^®^ 1.3.0.83 software (LI-COR Biosciences). Molecular masses were determined using the Chameleon^®^ Duo Pre-Stained Protein Ladder (LI-COR Biosciences). Experiments were performed in triplicate.

## 3. Results

*β2* ko mice develop a severe neurological phenotype and die during the third postnatal week. The retinal phenotype of *β2* ko mice is characterized by rapid apoptotic degeneration of photoreceptor cells [18,19]. In contrast, *β2*/*β1* ki mice have a normal lifespan and do not develop neurological symptoms or any other pathologies, except for a slowly progressive retinal dystrophy, demonstrating that the knocked-in β1 transgene partially compensates for the absence of the β2-subunit [20]. In the present study, we performed a detailed characterization of the retinal phenotype of the *β2*/*β1* ki mutant.

### 3.1. Neuroinflammation and Retinal Thinning

**Figure 1:**
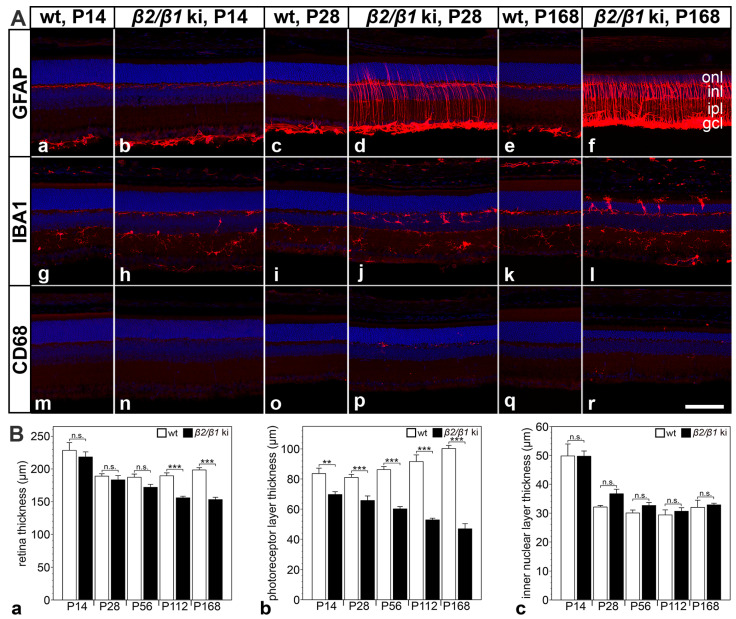
Neuroinflammation and retinal thinning in *β2*/*β1* ki mice. (**A**) Expression of GFAP (**Aa**–**Af**), IBA1 (**Ag**–**Al**), and CD68 (**Am**–**Ar**) in *β2*/*β1* ki and wild-type retinas at different ages. (**B**) Thickness of the total retina (**Ba**), the photoreceptor layer (**Bb**), and the inner nuclear layer (**Bc**) in *β2*/*β1* ki (filled bars) and wild-type retinas (open bars) at different ages. Each bar represents the mean value (±SEM) of six animals. CD68: cluster of differentiation 68; gcl: ganglion cell layer; GFAP: glial fibrillary acidic protein; IBA1: ionized calcium-binding adapter molecule 1; inl: inner nuclear layer; ipl: inner plexiform layer; ki: knock-in; onl: outer nuclear layer; P: postnatal day; wt: wild-type; n.s.: not significant, **: *p* < 0.01, ***: *p* < 0.001 by two-way ANOVA followed by a Bonferroni post hoc test. Scale bar: 100 µm.

Reactive astrogliosis, as evidenced by increased expression levels of glial fibrillary acidic protein (GFAP) in retinal astrocytes and Müller cells, was evident in *β2*/*β1* ki retinas at postnatal day (P) 28. GFAP expression levels further increased with increasing age of the mutants (Figure 1(Aa–Af)). The density of ionized calcium-binding adapter molecule 1 (IBA1)-positive microglial cells was increased in P14 *β2*/*β1* ki retinas compared with age-matched wild-type retinas (compare Figure 1(Ag,Ah)). In 168-day-old *β2*/*β1* ki mice, IBA1-positive cells had infiltrated the outer nuclear layer, and some microglial cells were located in the subretinal space (Figure 1(Al)). In addition, a few cluster of differentiation 68 (CD68)-positive microglia/macrophages were detectable in 28-day-old mutant retinas (Figure 1(Ap)) and were preferentially located in the subretinal space at P168 (Figure 1(Ar)). Wild-type retinas were devoid of CD68-positive cells at all ages analyzed (Figure 1(Am,Ao,Aq)). Taken together, these results demonstrate an early onset of neuroinflammation in the *β2*/*β1* ki retina.

The thickness of the *β2*/*β1* ki retina was similar to that of age-matched wild-type retinas until P56, but was significantly reduced in 4- and 6-month-old mutants when compared to the controls (Figure 1(Ba)). In contrast, photoreceptor layer thickness was significantly reduced in *β2*/*β1* ki mice as early as at P14 compared with wild-type mice. The thinning of the photoreceptor layer progressed with increasing age of the mutants. In 6-month-old animals, the thickness of the photoreceptor layer was reduced by more than 50% (Figure 1(Bb)). In contrast, the thickness of the inner nuclear layer of *β2*/*β1* ki retinas was not significantly different from that of wild-type retinas up to the latest age analyzed (Figure 1(Bc)). The results suggest that a progressive loss of photoreceptor cells is the major, and possibly the only, pathologic alteration in the *β2*/*β1* ki retina at the morphologic level.

### 3.2. Expression of Na,K-ATPase Subunits in Photoreceptor Cells

**Figure 2:**
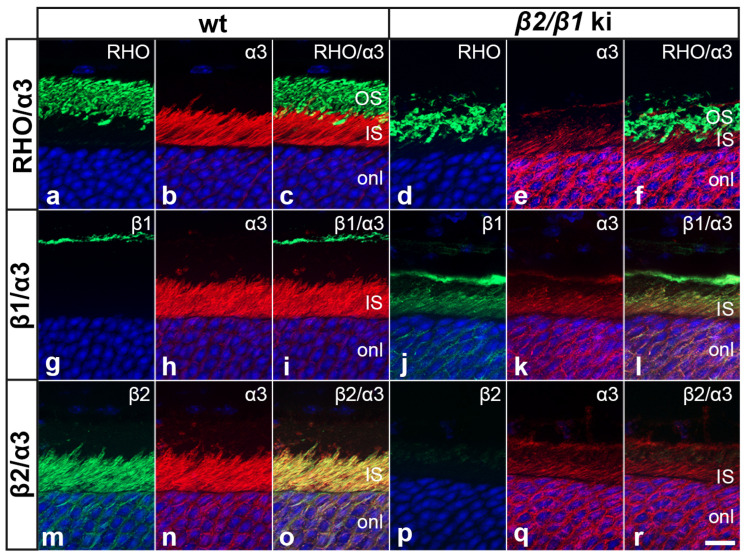
Expression of Na,K-ATPase subunits in wild-type and *β2*/*β1* ki photoreceptors. In wild-type photoreceptors, α3 is strongly expressed in the inner segments (**a**–**c**) and co-localized with the β2-subunit (**m**–**o**), whereas in *β2*/*β1* ki photoreceptors, α3 is weakly expressed in the inner segments (**d**–**f**) and co-localized with the β1-subunit (**j**–**l**). Inner segments of wild-type and *β2*/*β1* ki photoreceptors are β1- and β2-negative, respectively (**g**–**i** and **p**–**r**, respectively). IS: inner segments; ki: knock-in; OS: outer segments; RHO: rhodopsin. Scale bar: 10 µm.

We next analyzed the expression of the different β-subunits in photoreceptors of adult *β2*/*β1* ki and wild-type retinas. In wild-type retinas, β2 was strongly expressed in photoreceptor cells (Figure S1a,b). β2 immunoreactivity was also observed in the outer plexiform layer, and throughout the inner nuclear and inner plexiform layers (Figure S1). The mutant retinas were β2-negative, as expected (Figure S1c,d). In wild-type mice, expression of β1 was predominantly observed in the outer and inner plexiform layers and the nerve fiber layer, whereas the photoreceptor cells were β1-negative (Figure S1e,f). In contrast, β1 was weakly expressed in *β2*/*β1* ki photoreceptor cells (Figure S1g,h). Double immunostaining with antibodies against rhodopsin (RHO) showed that β2 in wild-type photoreceptors and β1 in mutant photoreceptors were restricted to the photoreceptor inner segments and absent from the outer segments (Figure S2). The predominant catalytic Na,K-ATPase subunit in photoreceptors, the α3-subunit, was strongly expressed in the inner segments of wild-type photoreceptors, but only weakly in the inner segments of mutant photoreceptors (Figure 2). In wild-type mice, α3 was co-localized with β2 (Figure 2m–o), consistent with previous reports [10,28]. In contrast, in mutant photoreceptors, α3 was co-localized with the knocked-in β1 transgene (Figure 2j–l). The results show that *β2*/*β1* ki photoreceptors express an α3β1 isozyme, albeit at low levels compared to the α3β2 isozyme expressed in wild-type photoreceptors.

**Figure 3:**
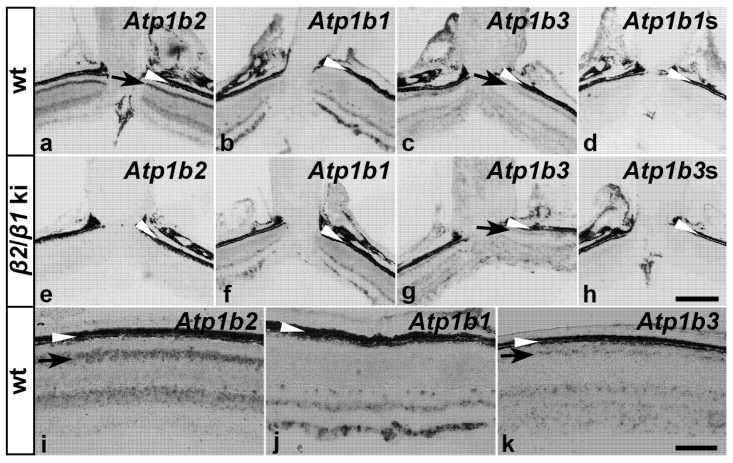
Expression of β-subunits in wild-type and *β2*/*β1* ki retinas. Expression of *Atp1b1*, *Atp1b2*, and *Atp1b3* transcripts in adult wild-type and *β2*/*β1* ki retinas. Black arrows in (**a**,**c**,**g**,**i**,**k**) indicate the localization of the indicated transcripts in the photoreceptor inner segments. The retinal pigment epithelium and choroid are indicated by white arrowheads (**a**–**k**). To control for signal specificity, *β2*/*β1* ki retinas were hybridized with an *Atp1b2* antisense probe (**e**) or an *Atp1b3* sense probe (*Atp1b3s*; (**h**)) and wild-type retinas were hybridized with an *Atp1b1* sense probe (*Atp1b1s*; (**d**)). ki: knock-in; wt: wild-type. Scale bar in (**h**) for (**a**–**h**): 200 µm, in (**k**) for (**i**–**k**): 100 µm.

Because of the lack of suitable anti-β3 antibodies for immunohistochemistry, we additionally analyzed the expression of β-subunits using nonradioactive *in situ* hybridization. Consistent with the immunohistochemical results, wild-type photoreceptors strongly expressed *Atp1b2* but not *Atp1b1* transcripts (Figure 3a,i and Figure 3b,j, respectively). We also observed a weak expression of *Atp1b3* mRNA (Figure 3c,k). In contrast, as expected, *β2*/*β1* ki photoreceptor cells did not express *Atp1b2* (Figure 3e). However, we were unable to detect transgenic *Atp1b1* mRNA in mutant photoreceptors (Figure 3f), despite clear evidence of β1 expression in the *β2*/*β1* ki photoreceptors at the protein level (Figure 2j, Figures S1 and S2), indicating that the expression level of the knocked-in transgene is below the detection level of our *in situ* hybridization method (see Discussion). Expression levels of *Atp1b3* transcripts in mutant retinas (Figure 3g) were similar to those observed in wild-type retinas (Figure 3c). No signals were detected in retinal sections hybridized with the *Atp1b1* (Figure 3d) or *Atp1b3* (Figure 3h) sense cRNA probe, or in *β2*/*β1* ki retinal sections hybridized with the *Atp1b2* antisense cRNA probe (Figure 3e).

### 3.3. Degeneration of Rod and Cone Photoreceptor Cells

**Figure 4:**
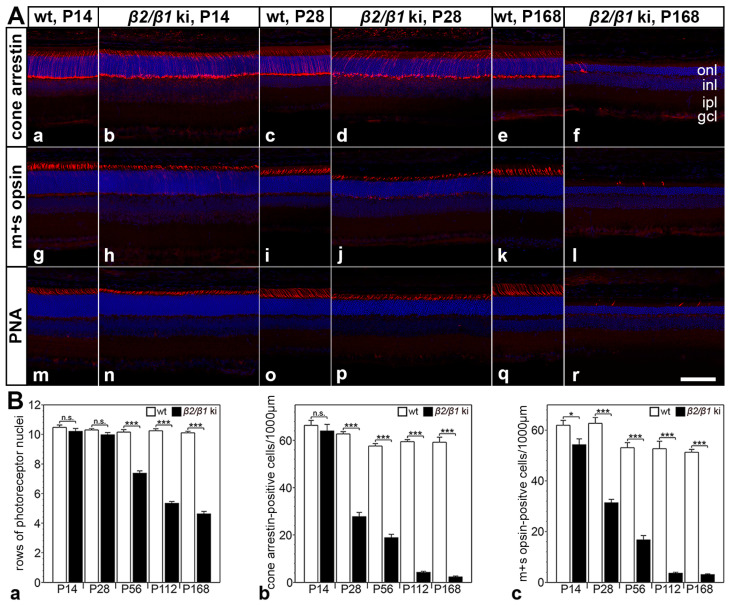
Photoreceptor cell degeneration in *β2*/*β1* ki mice. (**A**) *β2*/*β1* ki retinas show thinning of the outer nuclear layer (onl) and rapidly progressive loss of cone arrestin-positive (**Aa**–**Af**), m+s opsin-positive (**Ag**–**Al**), or PNA-labeled (**Am**–**Ar**) cone photoreceptor cells. Age-matched wild-type retinas are shown for comparison. (**B**) Number of rows of photoreceptor cell nuclei (**Ba**) and the density of cone arrestin- (**Bb**) or m+s opsin-positive (**Bc**) cones in *β2*/*β1* ki (filled bars) and wild-type retinas (open bars) at different ages. Each bar represents the mean value (±SEM) of six animals. n.s.: not significant, *: *p* < 0.05, ***: *p* < 0.001 according to two-way ANOVA followed by a Bonferroni post hoc test. gcl: ganglion cell layer; inl: inner nuclear layer; ipl: inner plexiform layer; ki: knock-in; onl: outer nuclear layer; P: postnatal day; PNA: peanut agglutinin; wt: wild-type. Scale bar: 100 µm.

Rods comprise ~97% of all photoreceptors in the mouse retina [37]. The pronounced thinning of the photoreceptor layer (Figure 1(Bb)) thus demonstrates a marked loss of rods within 6 months in the mutant retina. To quantify the progression of photoreceptor degeneration and to analyze whether cone photoreceptors are also affected in the mutant, we determined the number of rows of photoreceptor nuclei, and the number of cone arrestin- and m+s opsin-positive cones at different developmental ages (Figure 4). The number of rows of photoreceptor cell nuclei in the mutant retina was significantly reduced at P56 (Figure 4(Ba)). By P168, approximately 50% of the photoreceptor nuclei were lost, consistent with the ~50% thinning of the photoreceptor layer at this age. Because the mouse retina is rod-dominated, these data primarily reflect rod loss. A qualitative analysis of the sections stained with cone-specific markers revealed a similar density of cone arrestin, m+s opsin, and peanut agglutinin-positive cells in mutant and wild-type retinas at P14 (compare Figure 4(Aa,Ab,Ag,Ah,Am,An)). However, the inner and outer segments of cones were significantly shorter in mutant retinas compared to wild-type retinas (e.g., compare Figure 4(Am,An)). Notably, the number of cones in *β2*/*β1* ki retinas was markedly reduced as early as at P28 when compared with age-matched wild-type retinas (compare Figure 4(Ac,Ad,Ai,Aj,Ao,Ap)). In 6-month-old mutant retinas, cones were almost absent (Figure 4(Af,Al,Ar)). A quantitative analysis revealed a moderate but significant loss of m+s opsin-positive cones at P14 (Figure 4(Bc)). Cone degeneration progressed rapidly, resulting in a ~50% loss of cone arrestin-(Figure 4(Bb)) and m+s opsin-positive cells (Figure 4(Bc)) at P28. By P168, only about 5% of the cones remained (Figure 4(Bb,Bc)). Taken together, these observations demonstrate an early onset and rapid progression of cone photoreceptor degeneration in *β2*/*β1* ki mice.

### 3.4. Cell Types Other than Photoreceptors

**Figure 5:**
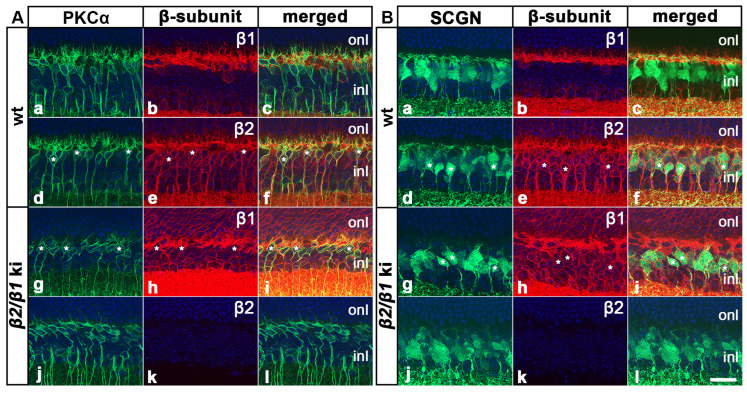
Expression of β1 and β2 in rod and cone bipolar cells. PKCα-positive rod bipolar cells and SCGN-positive cone bipolar cells in adult wild-type mice were β1-negative ((**Aa**–**Ac**) and (**Ba**–**Bc**), respectively) and β2-positive ((**Ad**–**Af**) and (**Bd**–**Bf**), respectively; some β2-positive cells are marked with white asterisks). In comparison, mutant rod and cone bipolar cells expressed β1 ((**Ag**–**Ai**) and (**Bg**–**Bi**), respectively; some β1-positive cells are marked with white asterisks) and were β2-negative ((**Aj**–**Al**) and (**Bj**–**Bl**), respectively). inl: inner nuclear layer; ki: knock-in; onl: outer nuclear layer; PKCα: protein kinase C alpha; SCGN: secretagogin; wt: wild-type. Scale bar: 20 µm.

We next examined whether cell types other than the photoreceptors were affected in the *β2*/*β1* ki retina. Our *in situ* hybridization experiments revealed the expression of *Atp1b2* transcripts throughout the inner nuclear layer of the wild-type retinas (Figure 3a,i) whereas *Atp1b1* transcripts were restricted to the upper and lower margins of this layer (Figure 3b,j), suggesting expression of β2 in bipolar cells. Therefore, we analyzed the expression of β1 and β2 in these interneurons using immunohistochemistry. Experiments showed that PKCα-positive rod bipolar cells and SCGN-positive cone bipolar cells in wild-type mice expressed the β2-subunit (Figure 5(Ad–Af) and Figure 5(Bd–Bf), respectively), but not the β1-subunit (Figure 5(Aa–Ac) and Figure 5(Ba–Bc), respectively). In comparison, rod and cone bipolar cells in *β2*/*β1* ki retinas were β2-negative as expected (Figure 5(Aj–Al) and Figure 5(Bj–Bl), respectively) and weakly expressed β1 (Figure 5(Ag–Ai) and Figure 5(Bg–Bi), respectively). Notably, rod and cone bipolar cells in mutant retinas extended only a few poorly developed dendritic processes into the outer plexiform layer when compared to wild-type retinas (Figure 5). In addition, we observed a weak signal with the *Atp1b2* antisense cRNA probe (Figure 3a,i) and a strong signal with the *Atp1b1* antisense cRNA probe (Figure 3b,j) in the retinal ganglion cell layer of wild-type mice, indicating coexpression of both β-subunits in RGCs.

**Figure 6:**
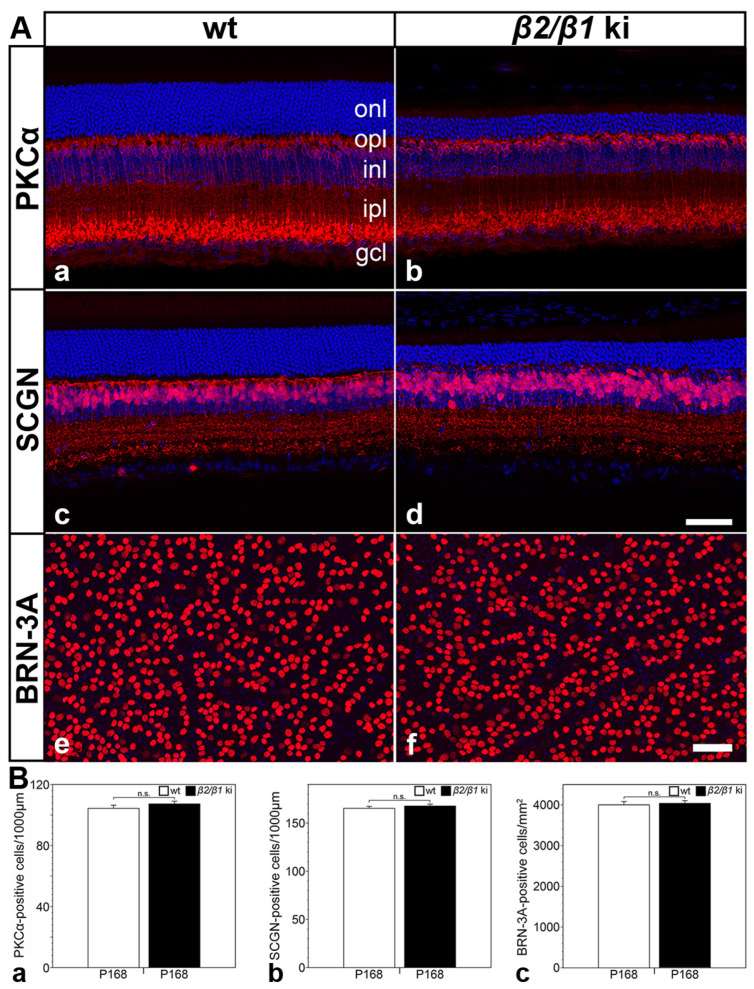
Normal number of rod and cone bipolar cells and ganglion cells in *β2*/*β1* ki retinas. Qualitative (**A**) and quantitative (**B**) analysis revealed similar densities of PKCα-positive rod bipolar cells (**Aa**,**Ab**,**Ba**), SCGN-positive cone bipolar cells (**Ac**,**Ad**,**Bb**), and BRN-3A-positive ganglion cells (**Ae**,**Af**,**Bc**) in wild-type and *β2*/*β1* ki retinas. Each bar in (**B**) represents the mean value (±SEM) of six animals. BRN-3A: brain-specific homeobox/POU domain protein 3A; gcl: ganglion cell layer; inl: inner nuclear layer; ipl: inner plexiform layer; ki: knock-in; onl: outer nuclear layer; opl: outer plexiform layer; P: postnatal day; PKCα: protein kinase C alpha; SCGN: secretagogin; wt: wild-type; n.s.: not significant according to the Student’s t-test. Scale bar in (**Ad**) for (**Aa**–**Ad**) and (**Af**) for (**Ae**,**Af**): 50 µm.

A qualitative analysis of the retinal sections or retinal flatmounts from 6-month-old mutant and age-matched wild-type mice revealed no obvious differences in the density of PKCα-positive rod bipolar cells (Figure 6(Aa,Ab)), SCGN-positive cone bipolar cells (Figure 6(Ac,Ad)), or BRN-3A-positive ganglion cells (Figure 6(Ae,Af)). A quantitative analysis confirmed similar numbers for each cell type in the retina of both genotypes (Figure 6B). The combined data suggest that rod and cone photoreceptors are the only cell types that degenerate in the *β2*/*β1* ki retina.

### 3.5. Expression of Retinoschisin, Kv2.1 and Kv8.2 in Wild-Type and Mutant Retinas

**Figure 7:**
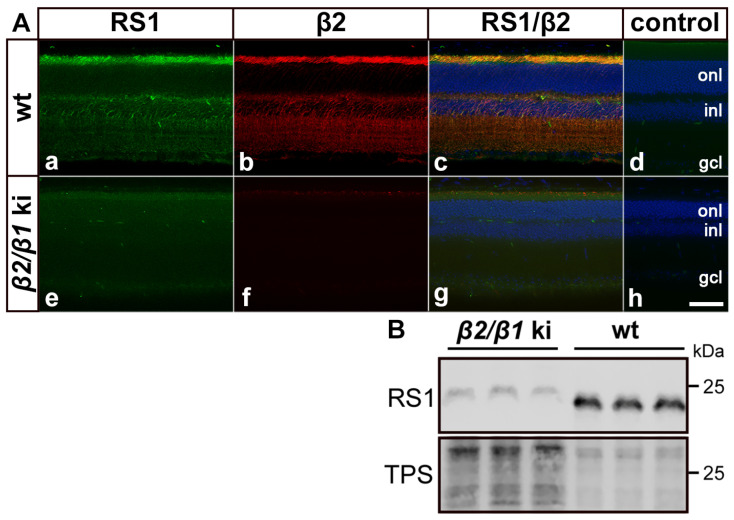
Expression of retinoschisin in mutant and wild-type retinas. In wild-type retinas, retinoschisin (RS1; (**Aa**)) and β2 (**Ab**) were co-localized (**Ac**) in the photoreceptor inner segments, outer plexiform layer, and cell bodies in the inner nuclear layer. In *β2*/*β1* ki retinas (**Ae**–**Ag**), retinoschisin was barely detectable and diffusely distributed (**Ae**). β2 was not detected in mutant retinas as expected (**Af**,**Ag**) is the overlay of (**Ae**,**Af**). As a negative control, sections were incubated with the secondary antibodies only (**Ad**,**Ah**). (**B**) Western blot analysis confirmed massively reduced levels of retinoschisin in mutant retinas when compared to wild-type retinas. Note that three-fold more mutant than wild-type samples were loaded. gcl: ganglion cell layer; inl: inner nuclear layer; ki: knock-in; onl: outer nuclear layer; RS1: retinoschisin; TPS: total protein stain; wt: wild-type. Scale bar: 50 µm.

Retinoschisin (RS1) interacts with the β2-subunit of the Na,K-ATPase, and RS1 protein levels are strongly reduced in the retina of *β2* ko mice [27,28,38,39]. Therefore, we investigated the expression of RS1 in adult wild-type and *β2*/*β1* ki retinas using immunohistochemistry and Western blotting. In wild-type retinas, RS1 and β2 were distributed in a similar pattern. Both proteins were strongly expressed in the photoreceptor’s inner segments and were also detected in the outer plexiform layer, on cell bodies located in the inner nuclear layer, and weakly in the inner plexiform layer (Figure 7(Aa–Ac)). In contrast, RS1 protein levels were massively reduced in *β2*/*β1* ki retinas when compared to the wild-type retinas (compare Figure 7(Aa,Ae)). Furthermore, RS1 immunoreactivity was no longer associated with the cell surface of the photoreceptor inner segments and retinal interneurons in the inner nuclear layer, but instead was diffusely distributed throughout the mutant retina (Figure 7(Ae)). Western blot analysis confirmed massively reduced levels of RS1 in mutant retinas (Figure 7B). A quantitative analysis of immunoreactive bands revealed a more than 20-fold decrease in the amount of RS1 protein in *β2*/*β1* ki retinas compared to wild-type retinas. Taken together, these results are in line with previous observations in *β2* ko retinas [28]. In addition, it has recently been shown that the voltage-gated potassium (Kv) channel subunits Kv2.1 and Kv8.2 interact with the Na,K-ATPase α3β2 in photoreceptors and that the expression of both subunits is significantly down-regulated in the photoreceptor inner segments of the *β2* ko mouse [29]. In accordance with these observations, an immunohistochemical analysis of *β2*/*β1* ki retinas revealed significantly reduced expression levels of Kv2.1 and Kv8.2 in the inner segments of mutant photoreceptors when compared with wild-type photoreceptors (Figure S3).

## 4. Discussion

*β2* ko mice develop a severe neurological phenotype, exhibit rapidly progressive photoreceptor cell degeneration, and die prematurely during the third postnatal week [18,19]. *β2*/*β1* ki mice express a fusion protein consisting of 18 N-terminal amino acids of the β2-subunit and amino acids 14 to 304 of the β1-subunit instead of β2 [20]. Unlike *β2* ko mice, *β2*/*β1* ki mice have a normal brain morphology and normal longevity. The only pathology observed in *β2*/*β1* ki mice was a loss of photoreceptor cells, which, however, progressed at a significantly slower rate than in *β2* ko mice [19,20]. These results show that the knocked-in β1 transgene partially compensates for the absence of β2. Whether cone and rod photoreceptor cells were differentially affected and whether nerve cell types other than photoreceptor cells degenerated in the mutant retina were not examined in these studies.

Early-onset retinal pathology in *β2*/*β1* ki mice was indicated by an increased number of IBA1-positive microglial cells at P14, and by an increased expression of GFAP in astrocytes and Müller cells and the appearance of CD68-positive macrophages/microglial cells at P28. The onset of neuroinflammation correlated with thinning of the outer nuclear layer, which was evident at P14 and progressed with increasing age of the *β2*/*β1* ki mice. An immunohistochemical analysis of Na,K-ATPase subunit expression revealed strong expression of an α3β2 isozyme in the inner segments of wild-type photoreceptors, consistent with other reports [10,22]. In contrast, *β2*/*β1* ki photoreceptor cells expressed an α3β1 isozyme. Notably, the expression of α3 and β1 proteins in mutant photoreceptors was weak compared to α3 and β2 in wild-type photoreceptors. In a previous study, we showed that transgenic *Atp1b1* transcripts in the brain of *β2*/*β1* ki mice amounted to only 10–20% of *Atp1b2* transcripts in the brain of wild-type mice, presumably due to the instability of the primary transcript [20]. Indeed, we were unable to detect transcripts of the knocked-in transgene in photoreceptors using *in situ* hybridization in a previous study [20] and in the present study using a different cRNA riboprobe. However, the expression level of the knocked-in β1 transgene was sufficient to significantly slow the degeneration of photoreceptors when compared to *β2* ko mice. Consistent with the fact that the correct intracellular trafficking and the integration of α-subunits into the cell membrane requires the presence of β-subunits, low-level expression of β1 resulted in weak expression of α3 in the inner segments of *β2*/*β1* ki photoreceptor cells [2,4]. Of interest in this context, the inner segments of photoreceptors in *β2* ko mice have been reported to be α3-negative [29].

Because of the absence of antibodies against β3 suitable for immunostaining, the expression of β-subunits was also analyzed using *in situ* hybridization. The experiments revealed low levels of *Atp1b3* transcripts in wild-type photoreceptors, confirming the results of an immunohistochemical study [10]. Weak expression of *Atp1b3* transcripts was also observed in *β2*/*β1* ki photoreceptors, potentially explaining the initially normal development of photoreceptor cells in *β2* ko mice [18,19].

To analyze whether rod and cone photoreceptors are differentially affected in *β2*/*β1* ki retinas, we determined the number of both photoreceptor types in wild-type and mutant mice. No significant loss of photoreceptor cells was observed in *β2*/*β1* ki retinas before P56. However, by P168, only about 50% of the photoreceptors remained, consistent with the ~50% thinning of the photoreceptor layer observed at this age. The results demonstrate progressive degeneration of rod photoreceptors, which comprise approximately 97% of all photoreceptors in the mouse retina [37]. Because significant photoreceptor loss became apparent relatively late at P56, we investigated whether the early neuroinflammatory response observed in the *β2*/*β1* ki retina was a result of an early-onset cone degeneration. Visualization of cones using three different markers consistently showed a normal number of cones at P14. Notably, the number of cones was significantly reduced at P28, and hardly any cones remained in the mutant retina at P168. A quantitative analysis revealed that approximately 50% of the cones were lost at P28, and only about 5% of the cones remained in 6-month-old mutants. Taken together, these results demonstrate a rapidly progressive loss of cones that is followed by a slowly progressive loss of rods, indicating that cones are more susceptible to Na,K-ATPase dysfunction than rods. This finding may be related to the fact that cones, unlike rods, do not saturate and, therefore, have to extrude Na^+^ ions that enter through cyclic nucleotide-gated channels in the outer segment even in bright light [40,41].

Mutations in *ATP1A1*, *ATP1A2*, or *ATP1A3* cause severe neurological disorders, such as Charcot-Marie-Tooth disease, complex spastic paraplegia, familial hemiplegic migraine type 2, rapid-onset dystonia-parkinsonism, alternating hemiplegia of childhood, or cerebellar ataxia, areflexia, pes cavus, optic atrophy, and sensorineural hearing loss (CAPOS) syndrome [42,43,44,45]. Of particular interest in the present context is a recent study that has identified a missense mutation (c.1772A > T, p.D591V) in exon 13 of the *ATP1A3* gene that co-segregated with the disease in a family with a clinically diagnosed autosomal dominant cone–rod dystrophy [23]. When this *ATP1A3* variant was ubiquitously expressed in mice on a wild-type background, the animals had apparently normal retinal morphology up to 3 months of age, as assessed by optical coherence tomography imaging and immunohistochemical analysis. Retinal function, as assessed by scotopic and photopic electroretinogram (ERG) recordings, was also unaffected at this age. However, when the ERG recordings were performed on 12-month-old mice, a-wave amplitudes were significantly reduced under photopic conditions, indicating impaired cone function [23]. Taken together, the results in patients and the mouse model are consistent with our findings in the *β2*/*β1* ki mutant, in that Na,K-ATPase dysfunction affects cones more than rods.

Similar to photoreceptor cells, rod and cone bipolar cells in wild-type mice strongly expressed β2, but not β1. In the *β2*/*β1* ki mutant, these interneurons weakly expressed the knocked-in β1 transgene. Unlike photoreceptors, however, we found comparable densities of rod and cone bipolar cells in 6-month-old wild-type and age-matched mutant retinas. The dendritic arbors of bipolar cells in the mutant retinas were less complex than those in the wild-type retinas, presumably due to the retraction of dendrites in response to progressive photoreceptor loss [46,47]. Contrary to an immunohistochemical study reporting that β3 expression is restricted to photoreceptor cells [10], we found weak and diffuse expression of *Atp1b3* transcripts throughout the inner nuclear layer of wild-type and mutant retinas, suggesting that bipolar cells express β3 in addition to β2. Indeed, the expression of β3 in these interneurons would explain why apoptotic cells in the *β2* ko mouse were confined to the photoreceptor layer until the premature death of the mutant during the third postnatal week [19]. In any case, our findings indicate that the knocked-in β1 transgene, possibly together with endogenously expressed β3, provides bipolar cells with sufficient Na,K-ATPase activity to survive into adulthood. The number of retinal ganglion cells, which express β1 and β2 [10], was also similar in wild-type and *β2*/*β1* ki mice. Taken together, the results indicate that the highly metabolically active photoreceptors are the only cell types affected in the mutant retina.

Retinoschisin (RS1), a 24 kDa discoidin domain-containing protein, is secreted as a homo–octameric complex that plays a critical role in maintaining the structural integrity of the retina. Loss-of-function mutations in the *RS1* gene cause X-linked retinoschisis (XLRS), a disease with a prevalence of 1:5000–1:25,000 that is characterized by the splitting of retinal layers, intraretinal cystic cavities, and a decreased b/a-wave amplitude ratio in scotopic ERG recordings, indicating impaired signal transmission from photoreceptor cells to bipolar cells [24,25,26,48]. RS1 and the α3β2 isozyme co-localize on the surface of the photoreceptor inner segments, in the outer plexiform layer, and on the cell surface of bipolar cells. Notably, RS1 has been shown to interact with the Na,K-ATPase α3β2 heterodimer, and that the isozyme is obligatory for RS1 anchoring to retinal membranes [27,28]. Indeed, the RS1 membrane association was lost and the RS1 protein levels were dramatically reduced in *β2* ko retinas [28]. Subsequent experiments using heterologous expression systems demonstrated that RS1 interacts specifically with β2, but not with α3 or any other Na,K-ATPase α- or β-subunit [38,39]. In line with these findings, we observed that RS1 was no longer associated with the cell surface of the *β2*/*β1* ki photoreceptor inner segments and bipolar cells that lack β2 but have low-level expression of an α3β1 isozyme.

XLRS animal models show progressive loss of photoreceptor cells (summarized in [49]). Intriguingly, the *Rs1h* ko mouse generated by the Weber group showed an early-onset apoptotic degeneration of photoreceptor cells with less than 50% of cones remaining in 2-month-old mutants [50,51,52], reminiscent of the retinal phenotype of *β2*/*β1* ki mice. Interestingly, treatment of Y-79 cells, human retinoblastoma cells that endogenously express α3β2, with recombinant RS1 resulted in decreased expression of the pro-apoptotic BCL-2 associated X protein (BAX). Treatment with recombinant RS1 also decreased caspase 3 activity in H_2_O_2_-stressed Y-79 cells. Furthermore, treatment of RS1-deficient retinal explants with recombinant RS1 significantly slowed the degeneration of cones and rods in RS1-deficient retinal explants [53]. Thus, it is tempting to speculate that, in addition to insufficient Na,K-ATPase activity, markedly reduced RS1 levels contribute to the progressive photoreceptor degeneration in *β2*/*β1* ki mice. Notably, despite an estimated 20-fold reduction of RS1 protein in *β2*/*β1* ki retinas, we did not observe other morphologic abnormalities reminiscent of those found in XLRS animal models, such as disorganized retinal layers and intraretinal cystic cavities [25,26,54].

The voltage-gated potassium channel subunit Kv2.1 and the electrically silent modulatory subunit Kv8.2, which is unable to form functional channels on its own but assembles with Kv2.1 to form functional heterotetrameric channels, are localized in inner segments of murine photoreceptors where they carry a large fraction of the outward dark current. Both Kv2.1-deficient mice, which additionally lack cell surface expression of Kv8.2, and Kv8.2-deficient mice, which express Kv2.1 homotetramers in the inner segments, exhibit dysfunction and degeneration of photoreceptors. [55,56,57,58]. In addition, mutations in *KCNV2*, the gene encoding Kv8.2, cause a retinal disorder in humans termed cone dystrophy with supernormal rod response (CDSRR) or KCNV2 retinopathy [30,31,59]. Interestingly, Kv2.1 and Kv8.2 have recently been identified as binding partners of the Na,K-ATPase α3β2 heterodimer [29]. Notably, protein levels of both Kv channel subunits were reported to be significantly reduced in the inner segments of *β2* ko photoreceptors, which also lack cell surface expression of α3 as a consequence of β2-deficiency [29]. Using immunohistochemistry, we also found significantly decreased Kv2.1 and Kv8.2 levels in the inner segments of the α3β1-expressing *β2*/*β1* ki photoreceptors. Whether and to what extent the dysregulated protein levels of the two Kv channel subunits affect the progression and severity of the retinal phenotype in *β2*/*β1* ki mice remains to be seen.

## 5. Conclusions

In summary, healthy mouse photoreceptor cells express high levels of β2, low levels of β3, and no β1 (Figure S4a). In photoreceptor cells of *β2* ko mice, expression of β3 alone resulted in insufficient Na,K-ATPase activity, leading to early-onset and rapidly progressive photoreceptor loss (Figure S4b) [18,19]. In *β2*/*β1* ki mice, expression of the knocked-in β1 transgene in addition to endogenous β3 slowed the photoreceptor degeneration observed in *β2* ko mice, demonstrating that the transgene partially compensates for the absence of β2 (Figure S4c). Cones were more severely affected than rods in *β2*/*β1* ki retinas, probably due to the higher energy demand of the former cell type. In addition, the results suggest that photoreceptor degeneration in *β2*/*β1* ki mice may be caused by a combination of insufficient Na,K-ATPase activity and significantly reduced expression levels of RS1, Kv2.1, and Kv8.2. Finally, *β2*/*β1* ki mice did not develop a schisis-like phenotype, despite the markedly reduced protein level and loss of cell surface association of retinoschisin. In conclusion, we consider the *β2*/*β1* ki mutant as an interesting animal model for studies aimed at developing treatment strategies for retinal diseases that preferentially affect cone photoreceptors.

## Figures and Tables

**Table 1:** Primary antibodies.

Antigen	Dilution	Supplier	Catalog Number
brain-specific homeobox/POU domain protein 3A (BRN-3A)	1:200 (IHC)	Santa Cruz Biotechnology Inc., Santa Cruz, CA, USA	sc-31984
cone arrestin	1:5000 (IHC)	Millipore, Temecula, CA, USA	AB15282
cluster of differentiation 68 (CD68)	1:1000 (IHC)	Bio-Rad-Laboratories, Kidlington, UK	MCA1957
glial fibrillary acidic protein (GFAP)	1:500 (IHC)	Dako Cytomation GmbH, Hamburg, Germany	Z0334
ionized calcium-binding adapter molecule 1 (IBA1)	1:200 (IHC)	Wako Chemicals GmbH, Neuss, Germany	019-19741
m opsin	1:500 (IHC)	Millipore	AB5405
protein kinase C alpha (PKCα)	1:500 (IHC)	Santa Cruz Biotechnology Inc.	sc-208
retinoschisin (RS1)	1:500 (IHC) 1:500 (WB)	Proteintech Group, Inc., Rosemont, IL, USA	24430-1-AP
rhodopsin (RHO) *	1:500 (IHC)	Abcam, Cambridge, UK	ab221664
rhodopsin (RHO) **	1:40,000 (IHC)	Sigma-Aldrich, Deisenhofen, Germany	O4886
s opsin	1:200 (IHC)	Santa Cruz Biotechnology Inc.	sc-14363
secretagogin (SCGN)	1:2000 (IHC)	BioVendor Research and Diagnostic Products, Eching, Germany	RD184120100
voltage-gated potassium channel subunit Kv2.1	1:2000 (IHC)	Antibodies Inc., Davies, CA, USA	75-014
voltage-gated potassium channel subunit Kv8.2	1:1100 (IHC)	Antibodies Inc.	75-435
α3-subunit	1:50 (IHC)	Proteintech Group, Inc.	10868-1-AP
β1-subunit	1:1000 (IHC)	[34]	N/A
β2-subunit	1:1000 (IHC)	[11]	N/A

IHC: immunohistochemistry; WB: Western blot; N/A: not applicable; *: used in Figure S2; **: used in Figure 2.

## Data Availability

The raw data supporting the conclusions of this article will be made available by the authors on request.

## Supplementary Materials

The following supporting information can be downloaded at: https://www.mdpi.com/article/10.3390/cells14120878/s1, Figure S1: Expression of the β2- and β1-subunit in adult wild-type and *β2*/*β1* ki retinas; Figure S2: Expression of β2- and β1-subunits in adult wild-type and *β2*/*β1* ki photoreceptor cells; Figure S3: Expression of Kv2.1 and Kv8.2 in adult wild-type and *β2*/*β1* ki photoreceptor cells; Figure S4: Summary of findings.
