# Supplementary material for: Atp1b2Atp1b1 Knock-In Mice Exhibit a Cone–Rod Dystrophy-Like Phenotype
Source: Cells. 2025 Jun 11;14(12):878. doi: 10.3390/cells14120878 (PMC12191355; doi:10.3390/cells14120878)
Supplement: Supplementary file 1 [file cells-14-00878-s001.zip › cells-3667344-supplementary.pdf]

Supplementary Materials

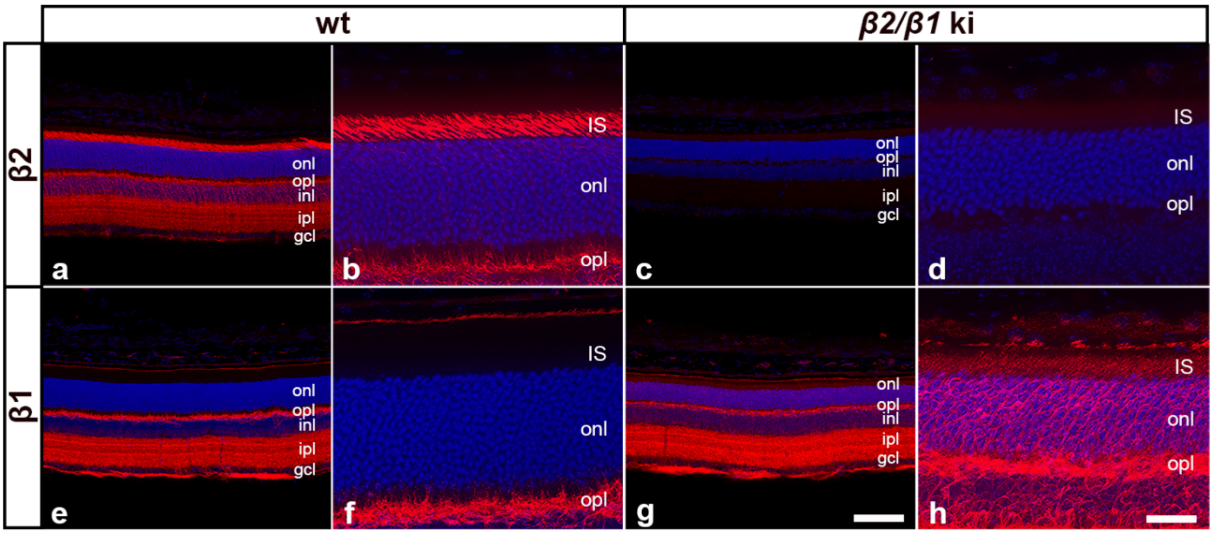

Figure S1. Expression of the β2- and β1-subunit in adult wild-type and *β2/β1* ki retinas. In wild-type retinas, β2 was strongly expressed in photoreceptors (**a**,**b**) and detectable in the outer plexiform layer (**a**,**b**), and the inner nuclear and inner plexiform layers (**a**). β1 expression was mainly restricted to the outer plexiform layer (**e**,**f**), the inner plexiform layer, and the nerve fiber layer (**e**). *β2/β1* ki retinas were β2 negative as expected (**c**,**d**). β1 in mutant retinas was weakly expressed in photoreceptors and detectable in the outer plexiform and inner nuclear layers (**g**,**h**), and the inner plexiform and nerve fiber layer (**g**). gcl, ganglion cell layer; inl: inner nuclear layer; ipl: inner plexiform layer; is: photoreceptor inner segments; ki: knock-in; onl: outer nuclear layer; opl: outer plexiform layer; wt: wild-type. Scale bar in (**g**) for (**a**,**c**,**e**,**g**): 100 μm, in (**h**) for (**b**,**d**,**f**,**h**): 50 μm.

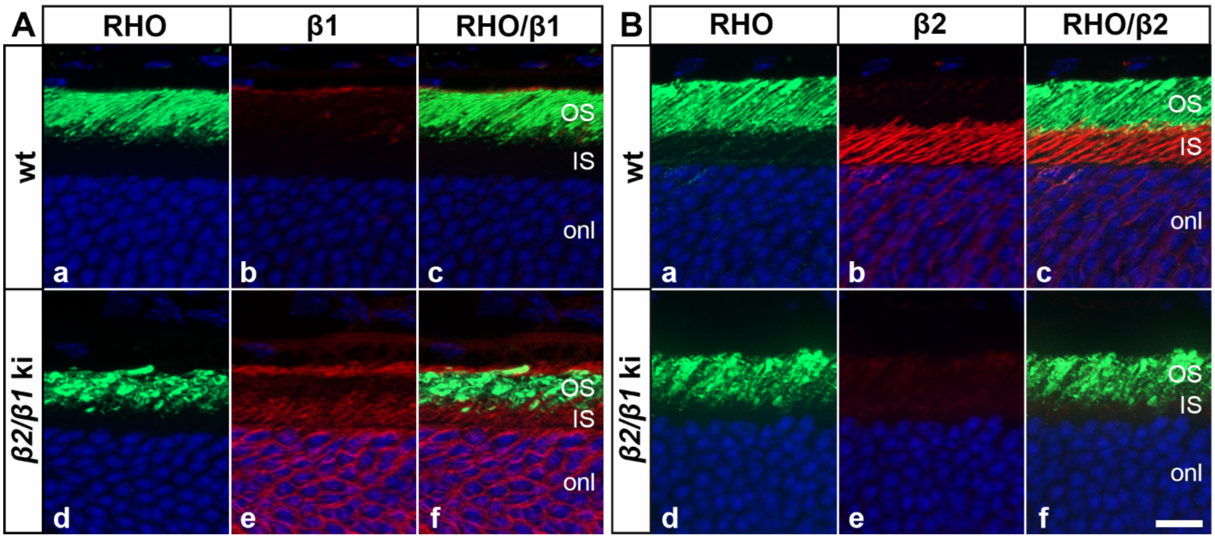

Figure S2. Expression of β2- and β1-subunits in adult wild-type and *β2/β1* ki photoreceptor cells. Double immunostaining with antibodies against rhodopsin and the β2- or β1-subunit showed strong expression of β2 in inner segments of wild-type photoreceptors (**Ba**-**Bc**) and weak expression of β1 in inner segments of mutant photoreceptors (**Ad**-**Af**). IS: inner segments; ki: knock-in; onl: outer nuclear layer; OS: outer segments; RHO: rhodopsin; wt: wild-type. Scale bar: 10 μm.

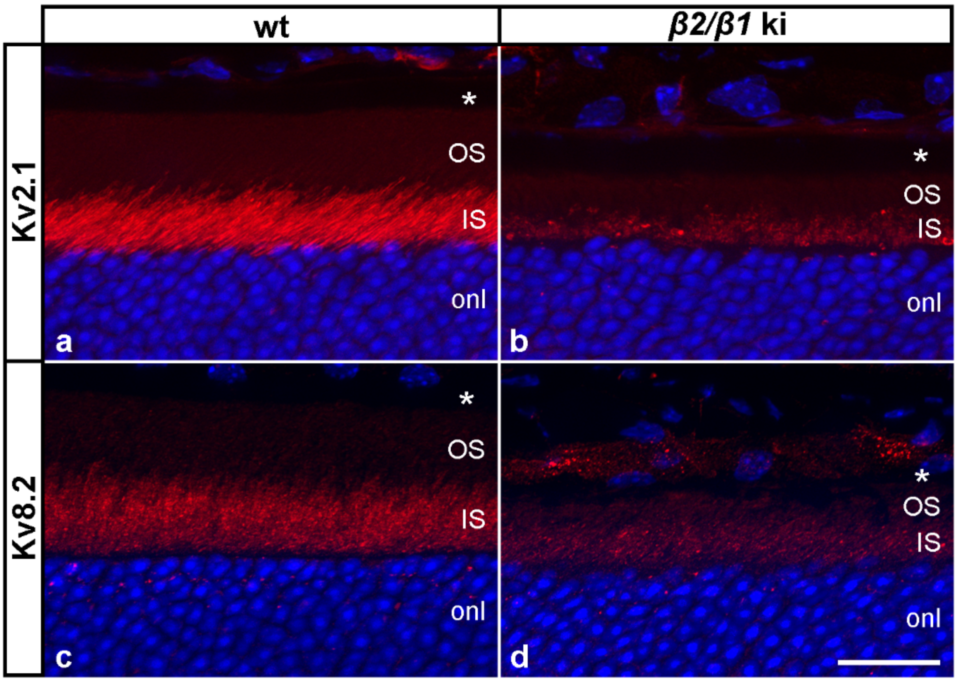

Figure S3. Expression of Kv2.1 and Kv8.2 in adult wild-type and *β2/β1* ki photoreceptor cells. Expression levels of Kv2.1 and Kv8.2 in inner segments of mutant photoreceptor cells (**b** and **d**, respectively) were significantly reduced when compared to wild-type photoreceptor cells (**a** and **c**, respectively). Asterisks mark the retinal pigment epithelium. IS: inner segments; ki: knock-in; onl: outer nuclear layer; OS: outer segments; wt: wild-type. Scale bar: 20 μm.

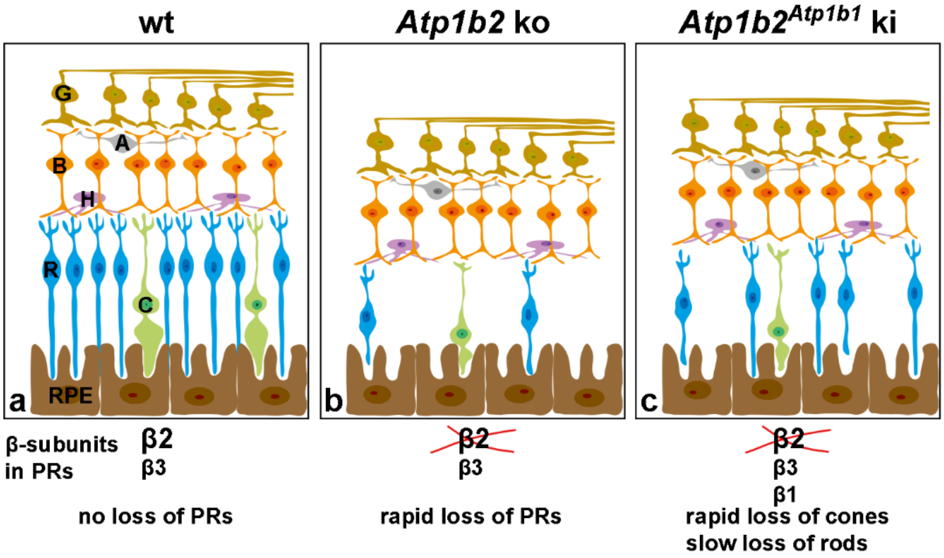

Figure S4. Summary of findings. Schematic representation of the expression of β-subunits in photoreceptor cells and the corresponding retinal phenotypes of wild-type (a), *β2* ko (b) and *β2/β1* ki mice. A: amacrine cells; B: bipolar cells; C: cones; G: ganglion cells; H: horizontal cells; PRs: photoreceptor cells; R: rods; RPE: retinal pigment epithelium.
